# Geometric Flow Control Lateral Flow Immunoassay Devices (GFC-LFIDs): A New Dimension to Enhance Analytical Performance

**DOI:** 10.34133/2019/8079561

**Published:** 2019-06-17

**Authors:** E. Eriksson, J. Lysell, H. Larsson, K. Y. Cheung, D. Filippini, W. C. Mak

**Affiliations:** ^1^Biosensors and Bioelectronics Centre, IFM-Linköping University, 58183 Linköping, Sweden; ^2^IKE-Linköping University, 58185 Linköping, Sweden; ^3^Optical Devices Laboratory, IFM-Linköping University, 58183 Linköping, Sweden

## Abstract

The nitrocellulose (NC) membrane based lateral flow immunoassay device (LFID) is one of the most important and widely used biosensor platforms for point-of-care (PoC) diagnostics. However, the analytical performance of LFID has limitations and its optimization is restricted to the bioassay chemistry, the membrane porosity, and the choice of biolabel system. These bottom neck technical issues resulted from the fact that the conventional LFID design principle has not evolved for many years, which limited the LFID for advanced biosensor applications. Here we introduce a new dimension for LFID design and optimization based on geometric flow control (GFC) of NC membranes, leading to highly sensitive GFC-LFID. This novel approach enables comprehensive flow control via different membrane geometric features such as the width (*w*) and the length (*l*) of a constriction, as well as its input angle (*θ*_1_) and output angle (*θ*_2_). The GFC-LFID (*w=0.5 mm, l=7 mm, θ*_1_*= 60*°, *θ*_2_*= 45*°) attained a 10-fold increase in sensitivity for detection of interleukin-6 (IL-6), compared with conventional LFID, whereas reducing by 10-fold the antibody consumption. The GFC-LFID detects IL-6 over a linear range of 0.1–10 ng/mL with a limit of detection (LoD) of 29 pg/mL, which even outperforms some commercial IL-6 LFIDs. Such significant improvement is attained by pure geometric control of the NC membrane, without additives, that only relaying on a simple high throughput laser ablation procedure suitable for integration on regular large-scale manufacturing of GFC-LFIDs. Our new development on GFC-LFID with the combination of facile scalable fabrication process, tailored flow control, improved analytical performance, and reduced antibodies consumption is likely to have a significant impact on new design concept for the LFID industry.

## 1. Introduction

Lateral flow immunoassay device (LFID) is one of the most successful biosensing platform technology, and nitrocellulose (NC) membrane based LFIDs are commercially represented by familiar home-based urine pregnancy tests. The technology supports numerous applications including clinical diagnostics, environmental assays, food and water safety, and pathogen testing [1–4]. LFIDs are technologically mature, feature a convenient shelf-life, and can naturally integrate basic passive sample conditioning. Furthermore, NC-LFID employs inexpensive and standardized materials, involving cost-effective manufacturing, and its configuration allows a seamless transition from research devices to scale production [1, 2, 5]. Once the bioassay chemistry and biolabel are defined [6–10], the NC membrane porosity is the only remaining variable to control the device performance and flow dynamic [11, 12]. Such limitation refers to the lack of an effective procedure to control the layout of the NC membrane at the core of the LFIDs. A practical solution would require not only enough resolution to operate within the confined geometry of classical 5 mm wide strips, but also avoidance of extensive damage and contamination of the NC membrane. Furthermore, such procedure should be compatible with integration in the large-scale manufacturing workflow, with minimum waste, avoiding process modifications. The relentless progress observed in paper analytical devices (PAD) in recent years [1, 13, 14] entails bespoke devices' layout, which leads to advanced configurations and flow control [15–19]. PAD rely on the confinement of paper conduits within hydrophobic barriers [20–22], which cannot be directly transferred to the NC platform in the required conditions, and despite the industrial importance of LFID, less progress has been seen in this area.

**Figure 1:**
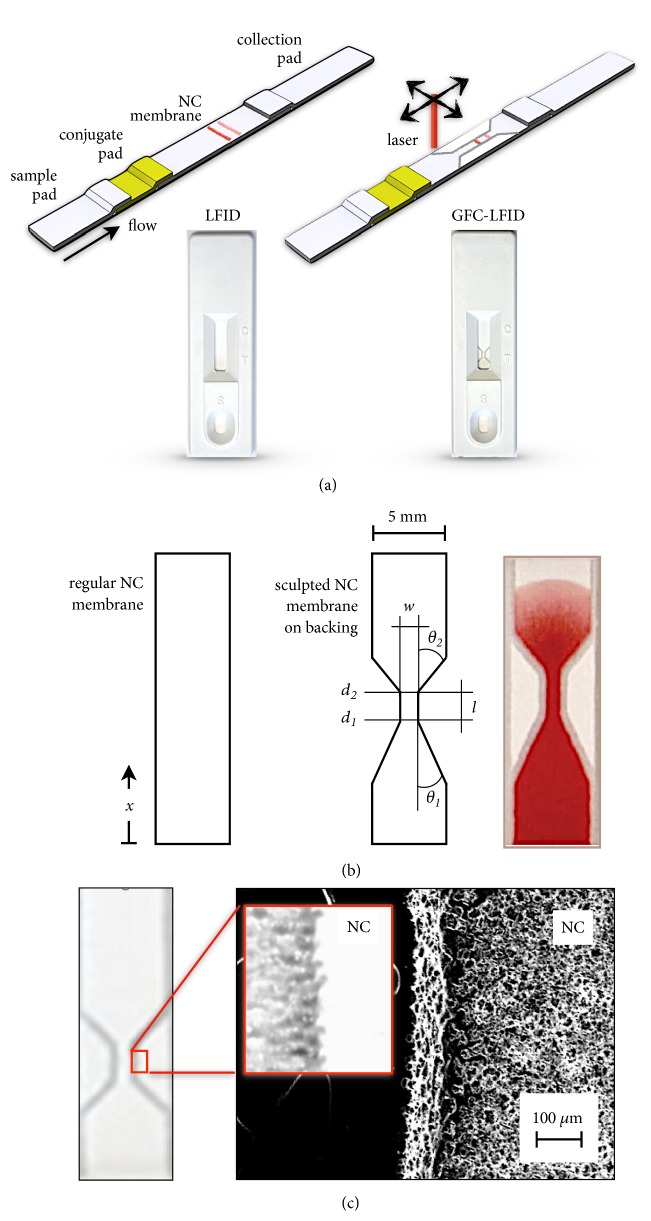
*Design and fabrication of GFC-LFIDs with various flow parameters.* (a) Scheme illustration of a classical LFID and GFC-LFID, and the corresponding devices. (b) 2D layout indicating the regular membrane geometry, with the flow axis* x*, and the geometric variables include the width (*w*) and the length (*l*) of a constriction, as well as its input angle (*θ*_1_) and output angle (*θ*_2_) in this study, along with an image of the actual GFC-LFID NC membrane. (c) Magnification of the laser ablated NC membrane of a GFC-LFID and the SEM image of corresponding boundary illustrating the NC morphology is affected by the laser processing.

The most classical configuration of lateral flow tests comprises a sample pad, a conjugation pad, a NC membrane with defined porosity, and an adsorption pad, all cut into strips (typically 5 mm wide) (Figure 1(a)). The sample pad is used to collect the sample fluid and, in some cases, is also able to perform sample pretreatment, such as separation of red-blood cells from the assay sample. The collected sample is then passively driven through the conjugation pad, which is preloaded with secondary biolabels that are specific for the target analytes. The mixture then flows along a nitrocellulose membrane where the primary biorecognition molecules are immobilized to create a test zone, and the affinity assay is performed. Finally, the excess sample fluid is collected by the adsorption pad. Typical quantitative readout entails the evaluation of the test line (where the capture chemistry is immobilized in the NC membrane) intensity [2, 23]. In such configuration, conventional flow rates, defined by the NC membrane porosity and constant cross-section, indicate that the antigen spends only between 1 and 6s at the test line, forcing concentrations of capturing antibody up to 100 times higher than in a static enzyme linked immunosorbent assays (ELISA) [1]. A technique capable of configuring the NC layout within the standard fabrication workflow could thus enable controlling the flow at the test line, optimizing the time that the analyte spends on such region, and focusing the flow of antigens to this point. Figure 1(a) illustrates the design concept of the GFC-LFID and Figure 1(b) details the control variables in this study as well as the picture of an actual laser configured NC membrane layout. Following the legacy of paper fluidic device research [13–19], we investigated the effect of well-established geometric flow control parameters in the laser configured NC membrane systems. Accordingly, we characterized influence of the width (*w*) and length (*l*) of the constriction, as well as its input angle (*θ*_1_) and output angle (*θ*_2_), on the flow regime and analytical performance. The resulting geometric flow controlled LFIDs are henceforth referred to as GFC-LFID (*w, l, θ*_1_, *θ*_2_).

## 2. Results and Discussions

### 2.1. Design and Fabrication of Geometric Flow Control Lateral Flow Immunoassay Devices (GFC-LFIDs) with Various Flow Parameters

Achieving such geometry with standard PAD fabrication is well established, but methods such as solid wax-based ink printing that are effective in paper do not translate equally well into NC membranes in confined spaces. Here instead, we entirely avoid hydrophobic barriers by direct laser ablation of the NC membrane in the desired layout [24]. Precise cutting at 1000 ppi and beyond can be produced with regular laser engravers; however, simply cutting the material is not enough to secure the integrity of the fragile NC membranes. With the current approach, the NC membrane integrity is secured by the backing substrate and only the confining space is selectively removed without damaging the active part of the NC membrane or the backing substrate.

NC membranes are geometrically sculpted by regulating the intensity of the laser engraver (40 W CO_2_ laser operating in raster mode at 1000 ppi, set at 40% power). Figure 1(c) shows an optical image of the laser configured NC membrane edge and the corresponding scanning electron microscope (SEM) image of a GFC-LFID device after laser ablation. A relatively smooth edge at the laser engraved NC membrane boundary is observed, whereas the nonaffected NC membrane remains intact keeping its original porosity. Using this technique, layouts with various widths from 0.2 mm to 4 mm (i.e., resolution of 200 *μ*m) were achieved ([Supplementary-material supplementary-material-1]), which compares favorably with conventional wax printing in PAD. Colored dye flow along the sculpted membranes shows a consistent confinement within the defined layout, in all cases ([Supplementary-material supplementary-material-1]). From a practical perspective, the laser ablation step is the only additional process to the regular manufacturing workflow, which benefits from the standard configuration of NC on a backing substrate. Laser engravers, even the simple version used in this work, can produce hundredths of engraved membranes per hour, with arbitrarily diverse designs, without added chemicals, with minimum waste, and without additional consumables.

### 2.2. Geometric Flow Control Analysis of Laser Assisted Configured Membranes

**Figure 2:**
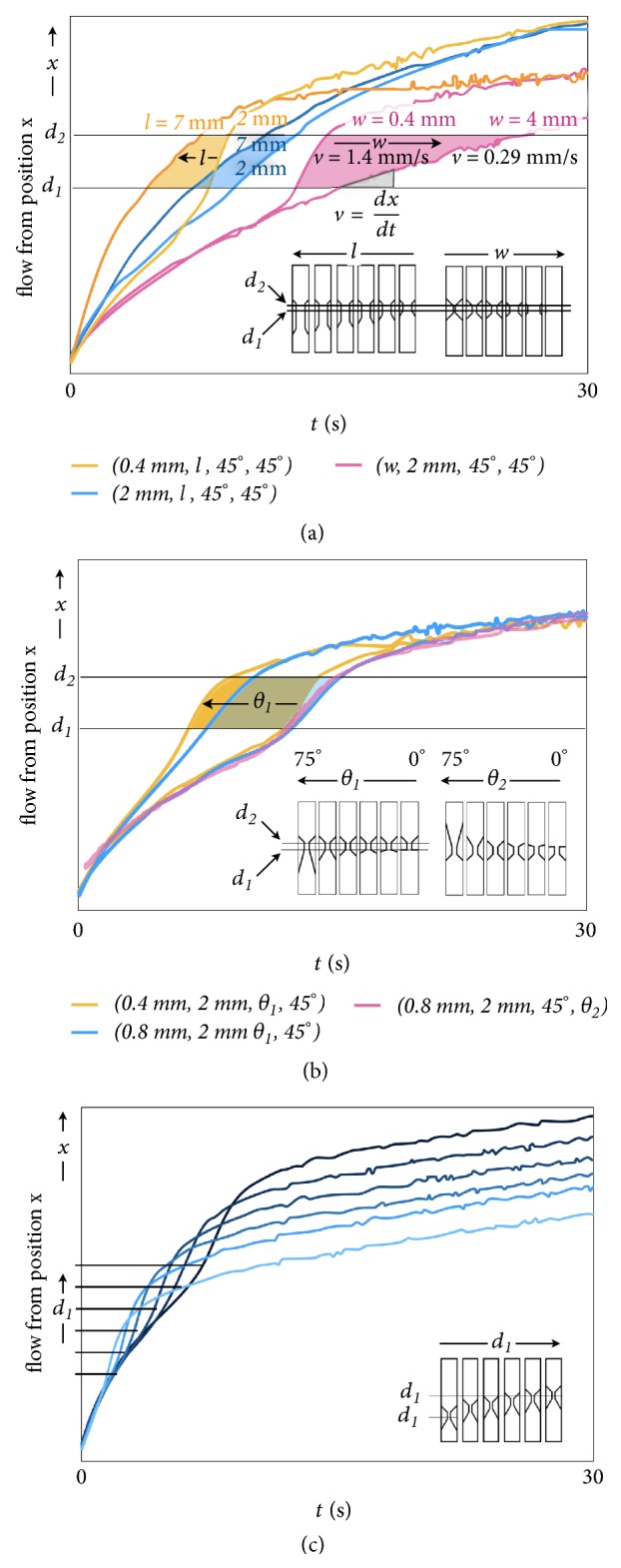
*Geometric flow control analysis of laser configured membranes.* (a) Flow front displacement of GFC-LFIDs (*w, l, 45*°*, 45*°) during 30 s interval recorded at 100 ms resolution as a function of the constriction length (*l*) and width (*w*) for fixed *θ*_1_ = *θ*_2_ = 45°. (b) Flow front displacement of GFC-LFIDs (*0.4, 0.8 mm, 2 mm, θ*_1_, *θ*_2_) during 30 s interval recorded at 100 ms resolution as a function of the constriction *θ*_1_ and *θ*_2_ between 0 and 75°, for constants *l* and *w*. (c) Flow front displacement at constant constriction width, angles, and length for different locations along the flow axis* x*.

Figure 2 collects the effect of the variables defining the NC layout on the flow behavior. Data is extracted from 25 fps videos of multiple geometries simultaneously captured (see [Supplementary-material supplementary-material-1] for details). Figure 2(a) shows the flow front displacement* vs.* time as a function of *w* ranging from 0.4 to 4.0 mm wide constrictions (GFC-LFID (*w, 2 mm, 45*°,* 45*°)), plotted at 100 ms resolution. Accordingly, the slope of these responses corresponds to the flow velocity (*v = dx/dt*) along the length of the membrane. In particular, between *d*_1_ and *d*_2_ the slope reflects the velocity within the constriction, where the detection takes place. For clarity, only the responses for *w* = 0.4 mm and 4 mm are displayed, whereas the intermediate responses in the constriction are indicated as the colored region. Further detail of the behavior of the intermediate responses can be found in [Supplementary-material supplementary-material-1], which shows a nonlinear decrease of the velocity with *w*. Regarding the volume flow rate (*q* = *v∗w∗h*, where *h* is the constant membrane thickness) the variation of *w* dominates over the velocity response resulting in an increasing flow rate with *w*.

The flow velocity can be regulated, within the constriction, from 1.4 to 0.29 mm/s (4.8x) for increasing widths (from 0.4 to 4 mm). The corresponding flow rates increase with *w*, according to the previous discussion, and span from 0.038 to 0.121 *μ*L/s (3.2x) for identical conditions ([Supplementary-material supplementary-material-1]). After the constriction, the flow decelerates in all cases, but more markedly for the narrowest constrictions, which suggest a strong limitation in the downstream flow in such conditions.

The performance of immunochromatographic assays is governed by the time it takes for the analyte solution to reach the test zone and for the analyte to interact at the test zone region of NC membrane. The GFC-LFID with a narrow constriction promotes a faster flow velocity, thus allowing an earlier sucking effect of the adsorption pad to drive the flow of sample solution. At the same time, the reduced flow rate at the GFC-LFID with narrower width provides longer incubation times per unit volume of analyte to interact with the assay membrane, facilitating the affinity interaction.

The effect of the constriction length (*l*) is also shown in Figure 2(a), for two different widths (0.4 and 2 mm). For clarity, only the limits of the response space are displayed (*l* = 2 and 7 mm), whereas the detailed effect of the *l* on the velocity and flow rate can be found in [Supplementary-material supplementary-material-1]. Figure 2(a) indicates that the flow velocity in the restricted region decreases with the width and length of such region, also showing a broader range of velocities for larger *w*. The flow velocity can be complementarily modulated with the constriction length* l*, as can be seen in Figure 2(a). For GFC-LFIDs (*0.4 mm, l, 45*°*, 45*°) the quantitative analysis shows a linear decrease of velocity between 1.38 and 0.57 mm/s (2.4x), and for *w* = 2 mm GFC-LFIDs (*2.0 mm, l, 45*°*, 45*°) between 0.61 and 0.48 mm/s (1.3x) ([Supplementary-material supplementary-material-1]). As previously analyzed, the flow rate magnitude (*q*) is dominated by the constriction width and increases with wider constrictions ([Supplementary-material supplementary-material-1]). Accordingly, the flow rate of GFC-LFIDs with varying *l* GFC-LFID (0.4 mm*, l, *45°, 45°) were between 0.072 and 0.030 *μ*L/s (2.4x), and GFC-LFIDs (*2.0 mm, l, 45*°*, 45*°) were between 0.143 and 0.113 *μ*L/s (1.3x) ([Supplementary-material supplementary-material-1]). Similarly, the flow rate shows a linear decrease with the constriction length.

The overall effects of the constriction input angle (*θ*_1_) and output angle (*θ*_2_) are displayed in Figure 2(b). The range of studied *θ*_1_ spans from 0 to 75° and the shown extreme responses in the figure enclose all the intermediate behaviors. Quantitative evaluations of velocity and flow are collected in [Supplementary-material supplementary-material-1]. The transit to the detection region is faster for larger *θ*_1_ and the spanned ranges (colored areas) partially overlap for different constriction widths (Figure 2(b)). The flow velocity could be regulated as a function of *θ*_1_, GFC-LFIDs (*0.4 mm, 2 mm, θ*_1_*, 45*°) between 1.28 and 0.81 mm/s (1.6x), and GFC-LFIDs (*0.8 mm, 2 mm, θ*_1_*, 45*°) between 0.87 and 0.64 mm/s (1.4x) ([Supplementary-material supplementary-material-1]), and the quantitative response shows a maximum at *θ*_1_ = 60°. The associated flow rates for GFC-LFIDs (*0.4 mm, 2 mm, θ*_1_*, 45*°) were between 0.069 and 0.044 *μ*L/s (1.6x), and for GFC-LFIDs (*0.8 mm, 2 mm, θ*_1_*, 45*°) were between 0.094 and 0.069 *μ*L/s (1.4x) ([Supplementary-material supplementary-material-1]).

The effect of *θ*_2_ is negligible as seen in the overlapping responses for the whole range of angles (Figure 2(b)), which is also quantitatively verified ([Supplementary-material supplementary-material-1]). Finally, the positioning of the constriction along the *x* axis contributes another degree of purely geometrical flow control, which essentially introduces an independent delay mechanism that does not affect other aspects of the flow (Figure 2(c)). When comparing the *w*,* l*, *θ*_1_, and *θ*_2_, it becomes clear that *w* offers the highest degree of modulation on the flow velocity (4.8x) and flow rate (3.2x), followed by the *l* and *θ*_1_, while the effect of *θ*_2_ is negligible.

Figure 4 summarizes the dominant combined effects of the geometry on the flow velocity. In this figure the color scale is an aid to the eye and highlights the identification of geometric configurations for defined goals. For instance, red circles are the conditions that maximize flow velocity.

### 2.3. GFC-LFIDs with Enhanced Signal and Sensitivity for Interleukin-6 (IL-6) Detection

**Figure 3:**
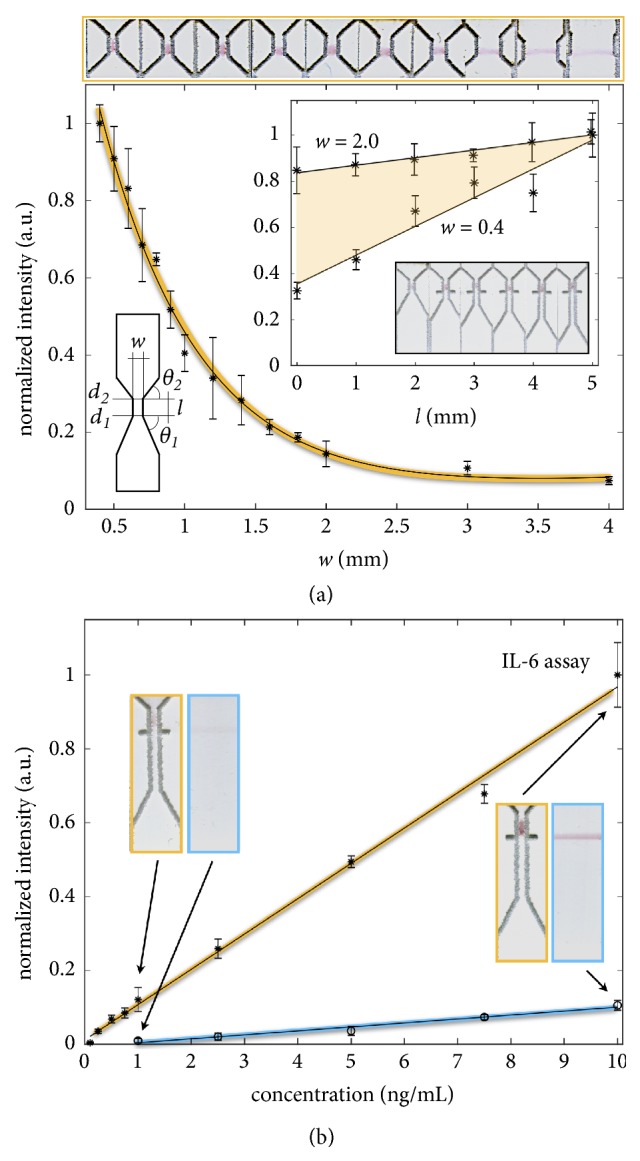
*Affinity analytical performance of GFC-LFIDs with enhanced signal and sensitivity*. (a) Capture efficiency evaluated by the intensity of the detection line for different constriction widths and lengths. The images are the crop of the actual simultaneously scanned samples used in the evaluation. Error bars correspond to a 95% confidence interval for samples measured in triplicate. (b) GFC-LFID (*0.5 mm, 7 mm, 60*°*, 45*°) shows a ~10-fold increase in sensitivity compared with the conventional LFID for detection of IL-6, while using only 1/10 of the antibody benefit to the small laser configured test zone region.

**Figure 4:**
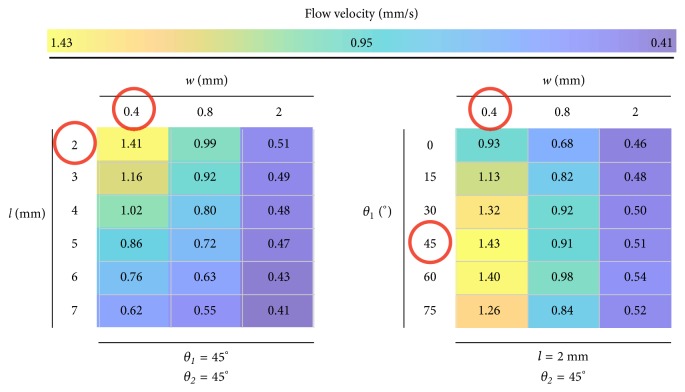
Flow velocity versus dominant combined geometric factors: restriction width (*w*), length (*l*), and input angle (*θ*_1_).

Complementarily to the flow analysis, the performance of the assay test zone for affinity binding was evaluated as a function of the two dominant geometric features: width and length of the constriction. AuNPs-*biotinyl-α*IL6ab conjugates were applied to the GFC-LFIDs (*w, 2 mm, 45*°*, 45*°) with width ranging from 0.4 to 4 mm, on which streptavidin were immobilized. The biotinylated AuNPs conjugates were captured by the streptavidin forming immunocomplexes at the test zone. The capture performances were evaluated by simultaneous scanning of entire geometric series, which are indicated as insets in Figure 3(a). The normalized intensity of the test zone shows an exponential increase of signal intensity with narrower constrictions, revealing a strong boost of the capture performance (13.5-fold). Complementarily, the effect on the length of the constriction (*l*) ranging from 2 to 7 mm were studied with the GFC-LFIDs (*0.4 mm, l, 45*°*, 45*°) and GFC-LFIDs (*2.0 mm, l, 45*°*, 45*°), respectively. The performance of the affinity interaction at the test zone region linearly increases with the length of the constriction (Figure 3(a) insert), and the effect is more pronounced for narrower constrictions. These observations are consistent with the flow analysis and highlight the fact that, purely by controlling the NC membrane layout, the capturing performance can be significantly improved. In this way, the restricted flow path concentrates the AuNPs at a smaller test zone region, while enabling the regulation of the flow rate (i.e., increasing flow velocity and decreasing flow rate) to an optimum pace for the affinity kinetics.

The studied behaviors were used to configure an GFC-LFID IL-6 assay for improved performance. IL-6 is a proinflammatory cytokine and is secreted by T cells and macrophages [25]. It plays an important role in various inflammatory responses and serves as an important biomarker for arthritis, inflammatory bowel disease, sepsis, cancer, and cardiovascular conditions [26, 27]. Figure 3(b) shows the performance of the GFC-LFID (*0.5 mm, 7 mm, 60*°*, 45*°) constriction, compared with the conventional 5 mm wide LFID. The GFC-LFID (*0.5 mm, 7 mm, 60*°*, 45*°) detects IL-6 over a linear range of 0.1–10 ng/mL, with a correlation coefficient of 0.996. The assay sensitivity was 844.8 ng/mL/pixel intensity and limit of detection (LoD) was 29 pg/mL (3×SD/sensitivity, n=3), respectively. In contrast, the conventional LFID configuration could only detect IL-6 between 1 and 10 ng/mL (no detectable signal below 1 ng/mL), with a sensitivity of 89.1 ng/mL/pixel intensity and a LoD of 1.29 ng/mL (3×SD/sensitivity, n=3). Images of the corresponding GFC-LFIDs and LFIDs were shown in [Supplementary-material supplementary-material-1]. The GFC-LFID (*0.5 mm, 7 mm, 60*°*, 45*°) shows a ~10-fold increase in sensitivity when compared with the conventional LFID for detection of IL-6, while using only 1/10 of the antibody due to the smaller test zone region, thus not only extending the range of the assay but also improving the economy of the solution by complementarily reducing the amount of antibody.

The enhanced analytical performance is attributed by the advanced geometric flow control of the laser configured NC membrane. During the assay, the IL-6 analyte molecules are constrained to pass through a smaller volume at the test zone of the laser configured NC membrane, therefore increasing the density of the immunocomplexes captured per unit volume at the narrowed test zone. In parallel, the narrowed test zone reduced the flow rate which provides a longer appearance incubation time for the affinity interaction to be occurred at the test zone region. It is worth noticing that this results at laboratory scale did not involve extensive optimization of the bioassay chemistry, or the source of antibodies, and that the observed increase in performance is purely merit of the geometry. Even in these conditions, the current assay is able to outperform some commercial counterparts of IL-6 lateral flow test with LoD of 50 pg/mL [28]* vs*. our developed IL-6 GFC-LFID with LoD of 29 pg/mL with a 42% lower LoD.

Concurrently, such result is attained without chemical modifications of the NC membrane or with procedures that in practice are not compatible with the large-scale manufacturing workflow. In contrast, laser configured NC membranes provide seamless integration in the regular fabrication procedure without adding materials to the LFID fabrication, which brings not only a new dimension to LFID optimization but also a cost-effective and practical innovation to the field.

## 3. Materials and Methods

### 3.1. Materials

Sample pad, conjugate pad, Hi-Flow 120 NC membrane, and adsorption pad were purchased from Millipore (Darmstadt, Germany). Gold nanoparticles (AuNPs) 40 nm was purchased from Arista Biologicals Inc. (Pennsylvania, USA). Biotinylated anti-interleukin-6 antibodies, primary anti-interleukin-6 antibodies, and recombinant human IL-6 were purchased from BioLegend Inc. (California, USA). Streptavidin was purchased from Thermo Fisher Scientific (Massachusetts, USA). Potassium carbonate (K_2_CO_3_), tris-(hydroxymethyl) aminomethane, bovine serum albumin (BSA), Tween 20, and polyethylene glycol (M.W. ~8000) (PEG) were purchased from Sigma Aldrich (Missouri, USA).

### 3.2. Preparation of Gold Nanoparticles (AuNPs) Biotinylated Anti-Interleukin-6 Antibodies (*Biotinyl*-*α*IL6ab) Conjugates

An aliquot of 5 mL of AuNPs solution (O.D. 1.64) was adjusted by addition of 0.1 M K_2_CO_3_ to pH 8.5. To the solution, 22.5 *μ*l 0.5 mg/mL biotinylated anti-interleukin-6 antibodies was added to the AuNPs and gently mixed for 5 min. Then, the AuNPs-*biotinyl-α*IL6ab conjugates were blocked with 1% PEG and 5% BSA overnight at 4°C. The resulting AuNPs-biotinyl-*α*IL6ab conjugates were harvested by centrifugation (16,000 g, 30 min) forming a pellet. From the centrifugated solution, the supernatant was discarded and the AuNPs-*biotinyl-α*IL6ab pellets were redispersed by addition of 10 mM Tris-HCl buffer (pH 7.0) to a concentration of O.D. 20 and stored at 4°C.

### 3.3. Laser Assisted Configuration of NC Membranes

Laser etching was performed with a HL40-5g Full Spectrum Laser LLC, a 40W CO_2_ laser engraving platform operating at 1000ppi resolution in raster mode. In order to exclusively remove the NC membrane, while minimizing damage of the membrane itself and the backing, different resolutions and laser power settings were tested. Since processing speed is paramount for large-scale processing, we only operated the platform at 100% laser speed and it was determined that a laser power of 40% at 1000 ppi attained the objective. Layouts for ablation can be created with diverse software packages and were produced as 300 dpi  .bmp files, as required, to be readable by the laser platform.

### 3.4. Assembling of LFIDs and GFC-LFIDs

The test zone on the NC membrane was prepared by dispending primary anti-interleukin-6 antibodies (0.5 mg/mL) or streptavidin (0.5 mg/mL) onto the NC membrane and laser etched NC membrane using the IsoFlow dispensing system at a dispensing rate of 0.1 *μ*L/mm (Arista Biologicals Inc., USA). The NC membrane was then dried in a desiccator overnight at room temperature. After drying, the absorption pad was assembled at the far end of the NC membrane downstream of the flow direction, and the conjugate pad and/or sample pad were assembled at the upstream of the NC membrane. Subsequently, the assembled LFIDs and GFC-LFIDs were cut into 5 mm wide strips by using an A-Point membrane cutter (Arista Biologicals Inc., USA). The resulting strips were stored in a desiccator at room temperature for future experiments.

### 3.5. Affinity Behavior of LFIDs and GFC-LFIDs

An aliquot of 40 *μ*L running buffer (10 mM Tris-HCl, 0.05% v/v Tween 20, pH 7.0) was mixed with 0.1*μ*L of the AuNPs-*biotinyl-α*IL6ab conjugates, and the solution mixture was then applied to the streptavidin coated LFIDs and GFC-LFIDs and allowed to run for 10 min. The assay was completed by further addition of 20 *μ*L of the running buffer. For quantitative evaluation, images of the GFC-LFIDs were recorded using a flatbed scanner (Epson Perfection V370 Photo). The color intensities of the test zones were analyzed with ImageJ software (Scion Corp., USA) and quantified as a function of pixel intensity.

### 3.6. GFC-LFIDs Assay for Interleukin-6 Detection

An aliquot of 40 *μ*L sample solution with various interleukin-6 (IL-6) concentrations ranging from 0.1 to 10 ng/mL was mixed with 1 *μ*L of the AuNPs-*biotinyl-α*IL6ab conjugates, and the solution mixture was then applied to the primary anti-interleukin-6 antibodies coated LFIDs and GFC-LFIDs and allowed to run for 10 min. The assay was completed by further addition of 20 *μ*L running buffer (10 mM Tris-HCl, 0.05% v/v Tween 20, pH 7.0). For quantitative evaluation, images of the LFIDs and GFC-LFIDs were recorded using a flatbed scanner (Epson Perfection V370 Photo). The color intensities of the test zones were analyzed with ImageJ software (Scion Corp., USA) and quantified as a function of pixel intensity. The images were converted to grey levels, and a region of interest along the strip axis intercepting the test line was established. The amplitude of the intensity profile measured with respect to the base line (membrane apart from the test line) was used to quantify the response intensity.

### 3.7. Flow Analysis

Videos were captured for flow analysis with a camera (NEX 5, Sony, Japan) at full resolution (1920x1080 pixels) and 25fps. The videos captured complete sets of conditioning variables, tested in identical conditions. Such videos were minimally repeated in triplicate for all the described conditions in this study. Qualitative figures (Figure 2) were automatically produced using a bespoke Matlab script. The videos were decomposed using Adapter 2.1.6 (Macroplant LLC), which created a numbered collection of  .jpg frames sampled at 10fps. The Matlab script enabled reading such collection of files and indicating regions of interest on every strip in the image, on which the automatic evaluation of the flow front was recorded. The code detected the flank of the flow front as a sharp drop in the blue channel of the image and recorded the corresponding position along the flow axes and time at 100 ms resolution. [Supplementary-material supplementary-material-1] summarizes such processing. Quantitative analysis of the flow regime was performed with Tracker (www.physlets.org/tracker) on the same videos. Such software requires manual introduction of tracking points in multiple frames, which allowed calibration and quantification of the flow velocity and volumetric flow rate.

## Supplementary Materials

Supplementary Materials Supplemental information includes five figures that can be found with this article online.
