# Supplementary material for: Geometric Flow Control Lateral Flow Immunoassay Devices (GFC-LFIDs): A New Dimension to Enhance Analytical Performance
Source: Research (Wash D C). 2019 Jun 17;2019:8079561. doi: 10.34133/2019/8079561 (PMC6750055; doi:10.34133/2019/8079561)
Supplement: Supplementary Materials — Supplemental information includes five figures that can be found with this article online. [file 8079561.f1.zip › 8079561.f1/8079561_SupplDesc.docx]

Supplementary Materials

Geometric flow control lateral flow immunoassay devices (GFC-LFIDs) – a new dimension to enhance analytical performance

E. Eriksson,^1^ J. Lysell,^1^ H. Larsson,^1^ K.Y. Cheung,^3^ D. Filippini,^2^* and W.C. Mak^1^*

^1^ Biosensors and Bioelectronics Centre, IFM-Linköping University, 58183, Linköping, Sweden

^2^ Optical Devices Laboratory, IFM-Linköping University, 58183, Linköping, Sweden

^3^ IKE-Linköping University, 58185, Linköping, Sweden

Correspondence should be addressed to D. Filippini; daniel.filippini@liu.se and W. C. Mak; wing.cheung.mak@liu.se

**Fig. S1. Sculpted NC membranes for LFIDs** with constriction widths (*w*) from 0.2 mm to 4 mm.

**Fig. S2.** **Colored contour plot summarizing the processing of each quantitative response in Fig. 2.** The image of each NC membrane in the video recording is analyzed along a region of interest (ROI) (blue line), which is collected for each time frame at 100ms resolution during a 90s interval. The plot captures the flow front profile and additional aspects as the gradient formation after the constriction. At *t_0_* the ROI collects a white membrane all along its length, and as the colored solution flows, at later times, the ROI captures its displacement. In this particular figure, already at 45s the position *d_2_* along the ROI is already saturated.

Fig. S3. Flow velocity and volume flow rate as a function of the constriction width. Error bars correspond to a 95% confidence interval, evaluated in triplicates.

**Fig. S4.** (A) **Flow velocity for different constriction lengths and widths**. Error bars correspond to a 95% confidence interval, evaluated in triplicates. (B) Volume flow rate for different constriction lengths and widths.

**Fig. S5.** (A) Flow velocity versus *θ_1_* for different restriction widths. (B) Volume flow rate versus *θ_1_* for different restriction widths. (C) Flow velocity versus *θ_1_* for different restriction widths. Error bars correspond to a 95% confidence interval, evaluated in triplicates.

**Fig. S6.** Images of the GFC-LFID and LFID after applying different concentration of IL-6.
