# Supplementary figures and images for: Geometric Flow Control Lateral Flow Immunoassay Devices (GFC-LFIDs): A New Dimension to Enhance Analytical Performance
Source: Research (Wash D C). 2019 Jun 17;2019:8079561. doi: 10.34133/2019/8079561 (PMC6750055; doi:10.34133/2019/8079561)

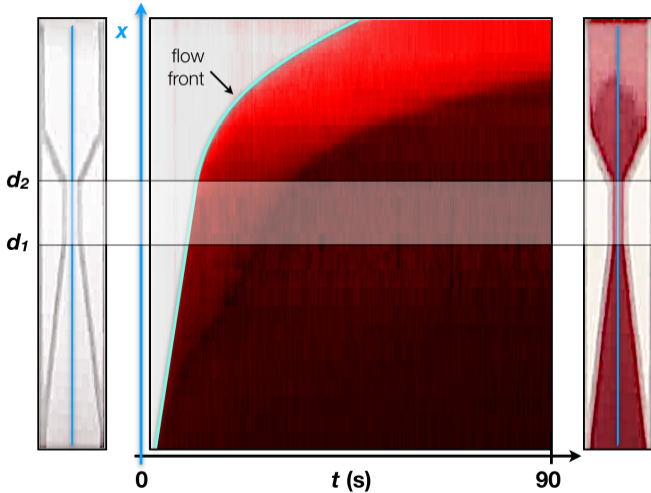

Supplement: Supplementary Materials — Supplemental information includes five figures that can be found with this article online. [file 8079561.f1.zip › 8079561.f1/figs_SI_02.pdf]

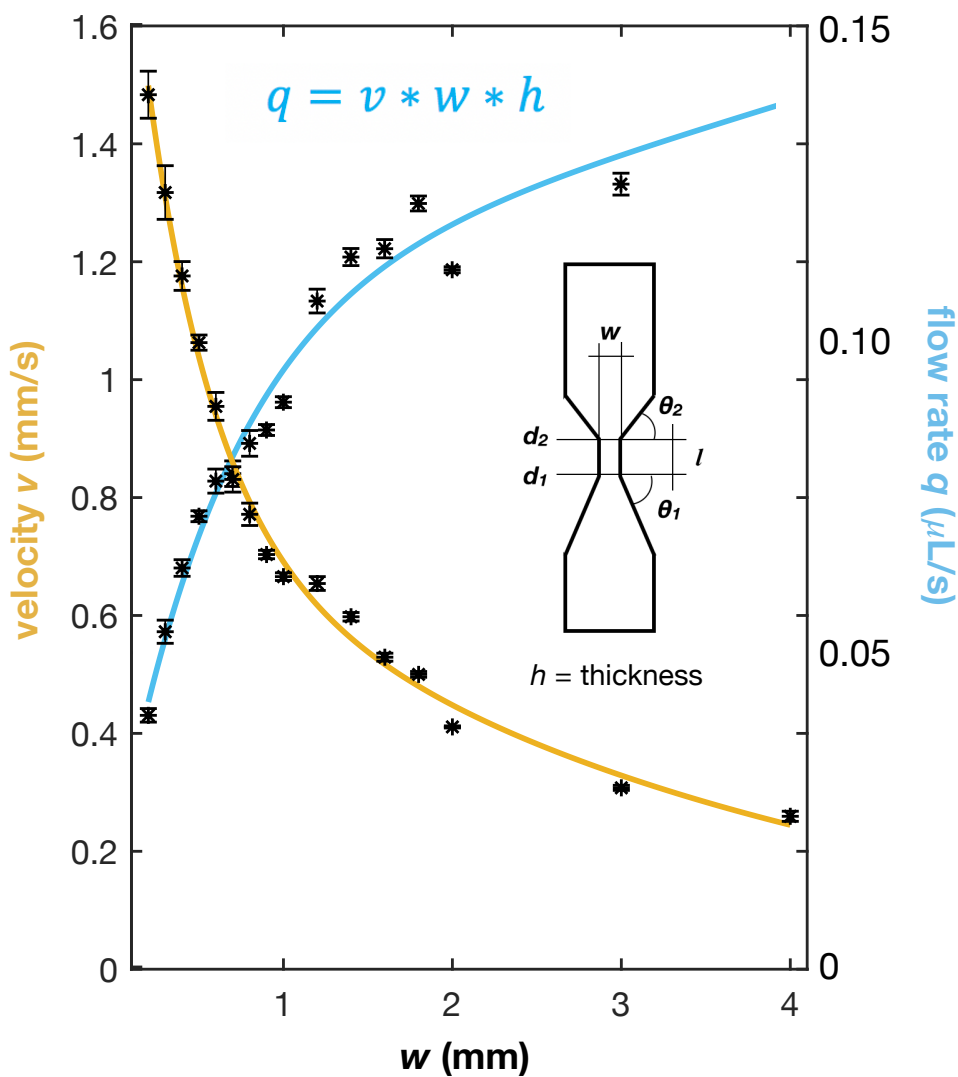

Supplement: Supplementary Materials — Supplemental information includes five figures that can be found with this article online. [file 8079561.f1.zip › 8079561.f1/figs_SI_03.pdf]

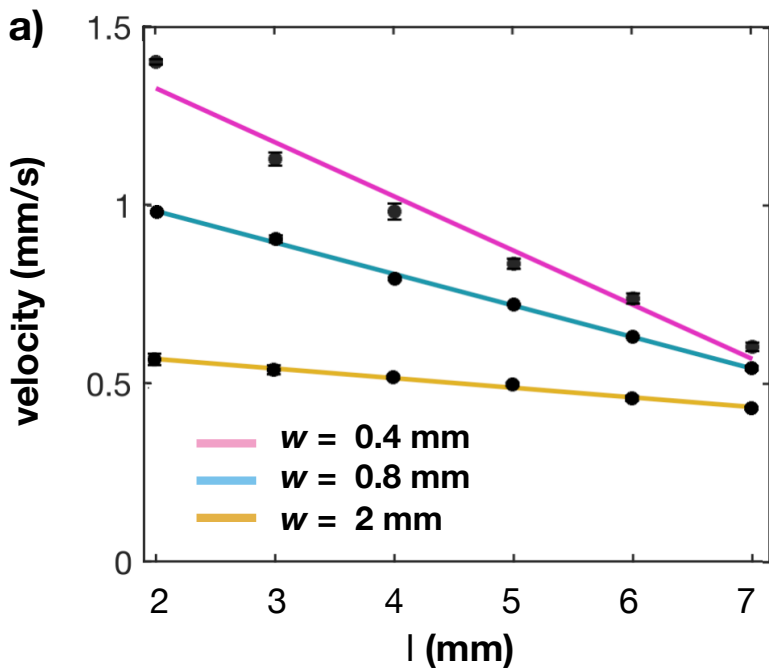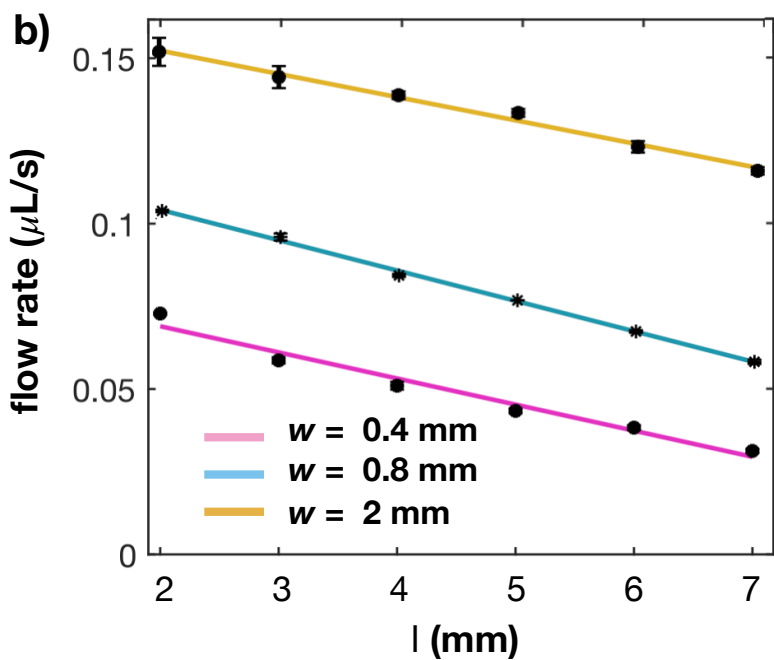

Supplement: Supplementary Materials — Supplemental information includes five figures that can be found with this article online. [file 8079561.f1.zip › 8079561.f1/figs_SI_04.pdf]

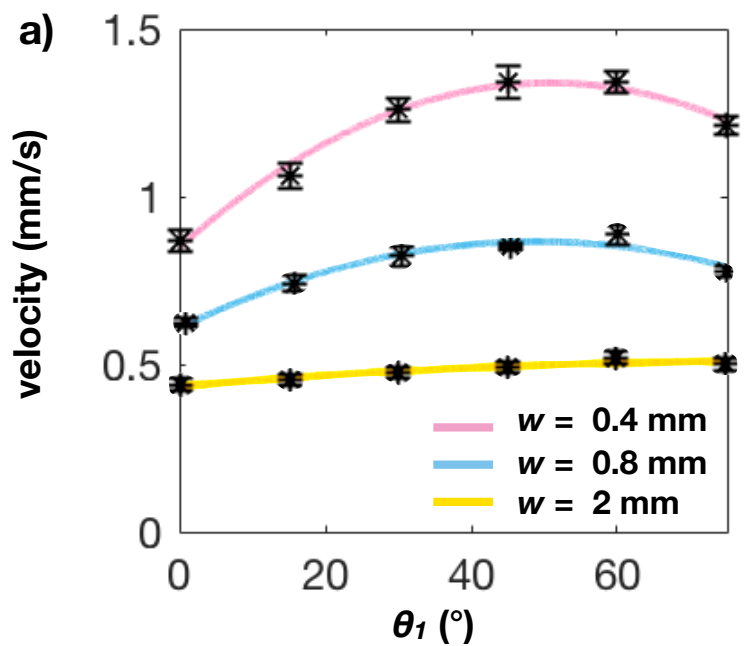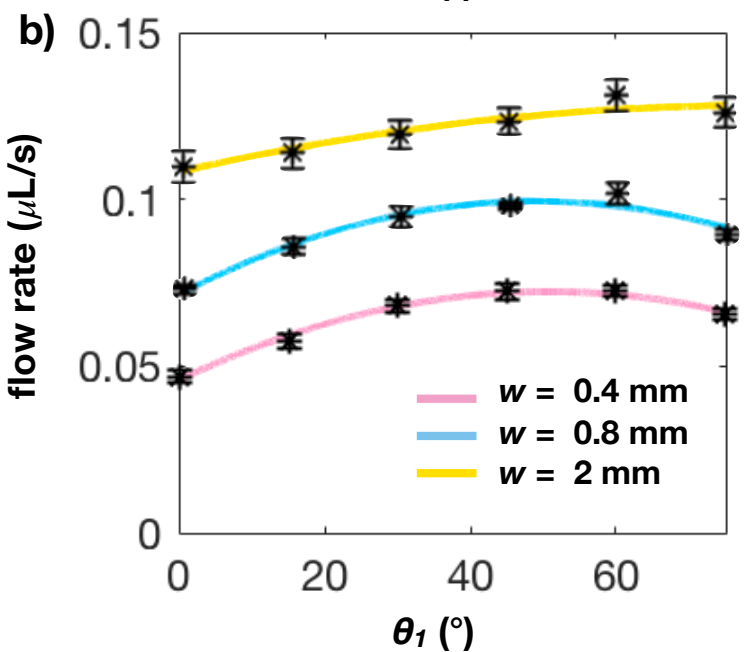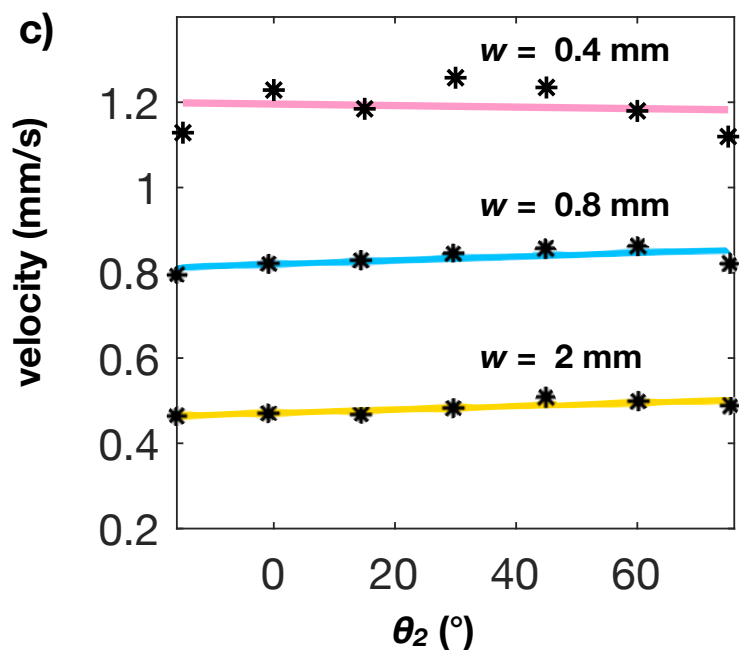

Supplement: Supplementary Materials — Supplemental information includes five figures that can be found with this article online. [file 8079561.f1.zip › 8079561.f1/figs_SI_05.pdf]

# GFC-LFIA

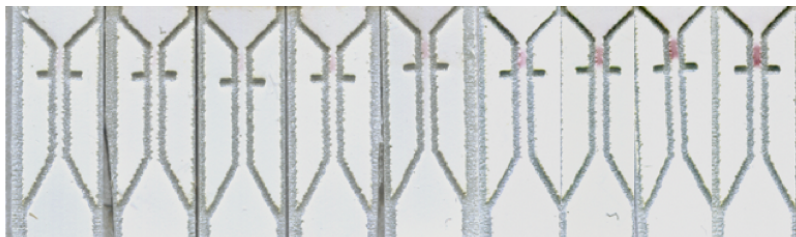

0 0.1 0.5 0.75 1.0 2.5 5.0 7.5 10

IL-6 concentration (ng/mL)

## LFIA

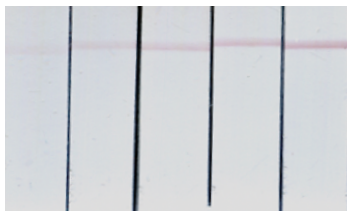

1.0 2.5 5.0 7.5 10

IL-6 concentration (ng/mL)

Supplement: Supplementary Materials — Supplemental information includes five figures that can be found with this article online. [file 8079561.f1.zip › 8079561.f1/figs_SI_06.pdf]

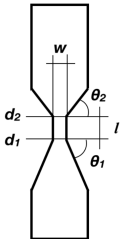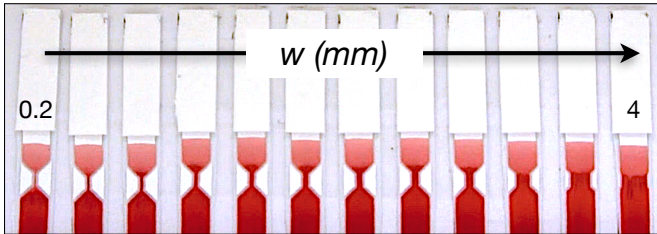

Supplement: Supplementary Materials — Supplemental information includes five figures that can be found with this article online. [file 8079561.f1.zip › 8079561.f1/fig_SI_01.pdf]
